# Combined effect of teriparatide and low-intensity pulsed ultrasound for nonunion: a case report

**DOI:** 10.1186/1756-0500-7-317

**Published:** 2014-05-27

**Authors:** Koji Nozaka, Yoichi Shimada, Naohisa Miyakoshi, Shin Yamada, Michio Hongo, Yuji Kasukawa, Hidetomo Saito, Hiroaki Kijima

**Affiliations:** 1 Department of Orthopedic Surgery, Akita University Graduate School of Medicine, 1-1-1 Hondo, Akita 010-8543, Japan

**Keywords:** Teriparatide, Low-intensity pulsed ultrasound (LIPUS), Nonunion, Bone quality

## Abstract

**Background:**

Low-intensity pulsed ultrasound is a pain-free therapy performed daily at home by the patient and has been shown to promote fracture healing. Teriparatide is a parathyroid hormone preparation that activates osteoblastic bone formation and is also reported to be effective in promoting bony union.

**Case presentation:**

We report the case of a 56-year-old Japanese male with a femoral shaft fracture who underwent intramedullary osteosynthesis nailing initially. He had no radiologic or clinical sign of healing 3 months later and low-intensity pulsed ultrasound was initiated at that time. He was reassessed in another 3 months, with evidence of mild bone consolidation but the fracture gap persisted. Subsequent treatment with human parathyroid hormone was initiated in combination with low-intensity pulsed ultrasound. Full fracture healing was present 6 months after beginning the combination low-intensity pulsed ultrasound and teriparatide. It is hypothesized that the potential additive effects of low-intensity pulsed ultrasound and teriparatide therapy ultimately triggered sufficient bone formation to support osseous union.

**Conclusion:**

The case reported herein is a femoral shaft atrophic nonunion in which traditional interventions failed. Successful fracture healing was finally achieved with low-intensity pulsed ultrasound and teriparatide therapy. This is the first reported case of diaphyseal nonunion with deterioration of bone quality in long bones resolved with teriparatide and low-intensity pulsed ultrasound.

## Background

The effect of low-intensity pulsed ultrasound (LIPUS) on the stimulation of bone formation in fracture and in cases of nonunion has been extensively studied [1]. However, there are cases in which bone union is not obtained even with the use of LIPUS, and it is difficult to judge the most appropriate timing for repeat surgery. A large physical, mental and social burden is placed on the patient when repeat surgery is needed. Teriparatide is a recombinant human parathyroid hormone [1–34] (PTH) preparation that is used in the treatment of osteoporosis, and is also reported to be effective in promoting bony union [2]. Teriparatide is effective because of its activation of osteoblastic bone formation [3]. Although both teriparatide and LIPUS have been found to accelerate fracture-healing processes [4], the effect of the combination of teriparatide and LIPUS in clinical bone fracture management is unclear.

## Case presentation

**Figure 1:**
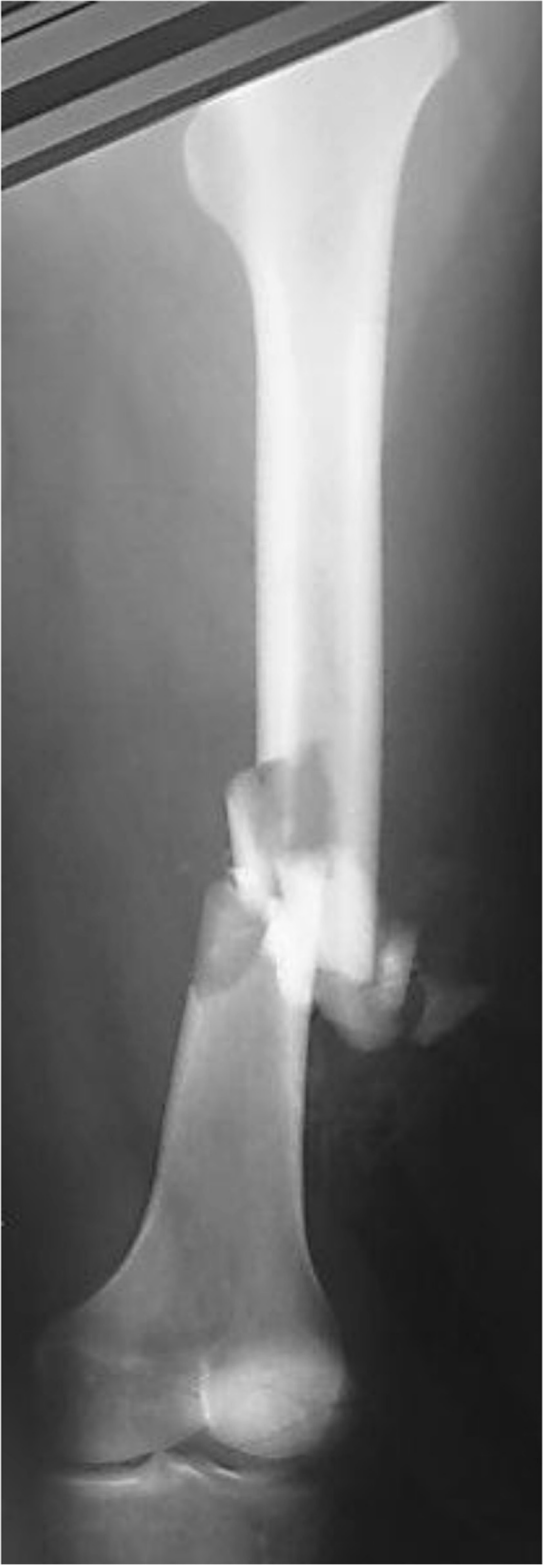
**Preoperative radiographs.** The femoral diaphyseal fracture (OTA 1, 32-C1.3).

**Figure 2:**
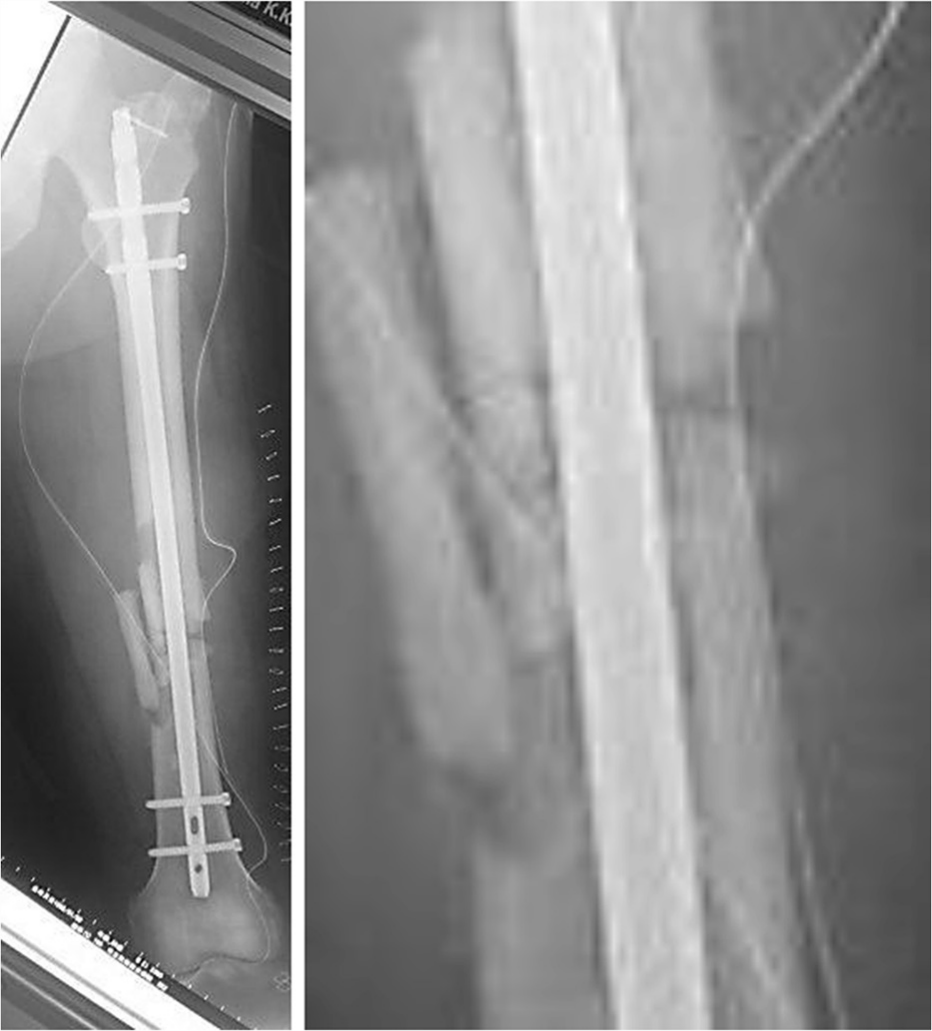
**Left, postoperative radiologic image of the femoral diaphyseal fracture (OTA 1, 32-C1.3) treated using interlocked intramedullary nailing.** Right, magnification of the image above.

**Figure 3:**
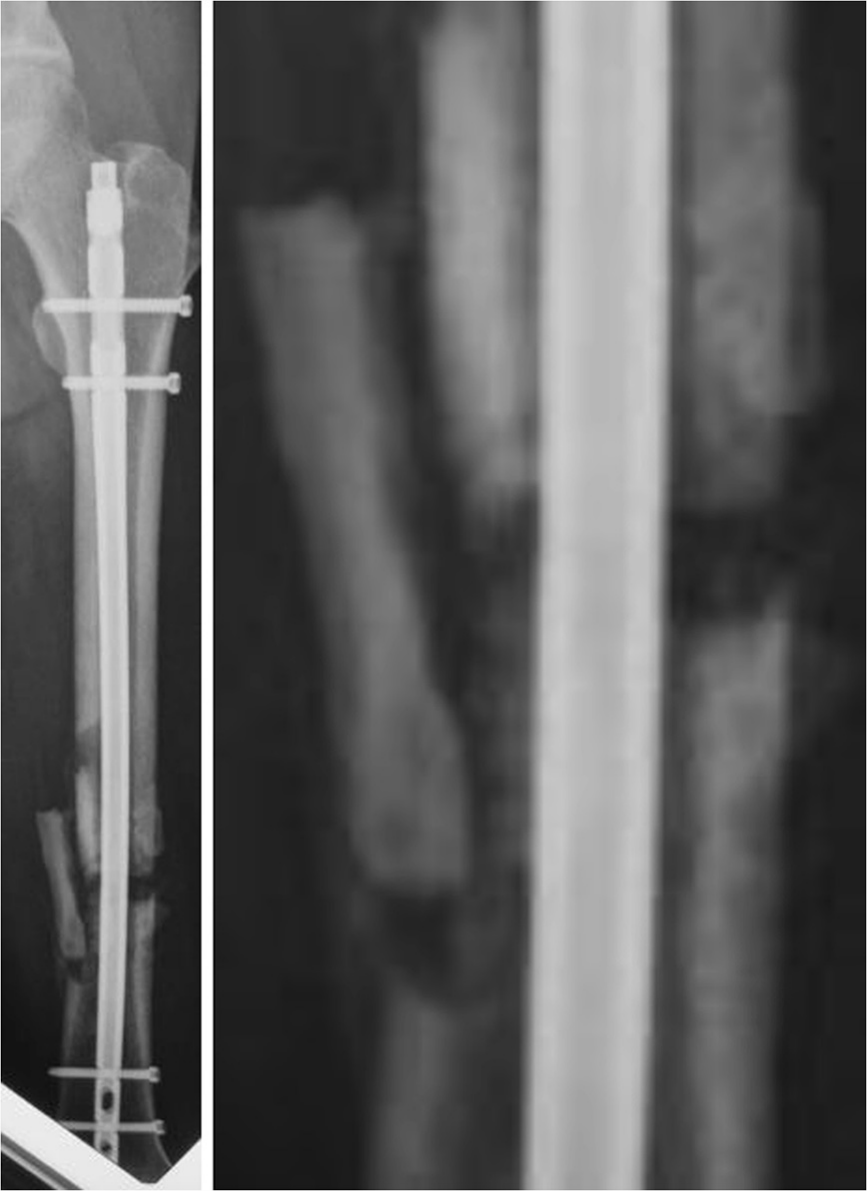
**Left, radiologic image at 3 months, showing no signs of healing but also no displacement.** LIPUS was started at this point. Right, magnification of the image above.

**Figure 4:**
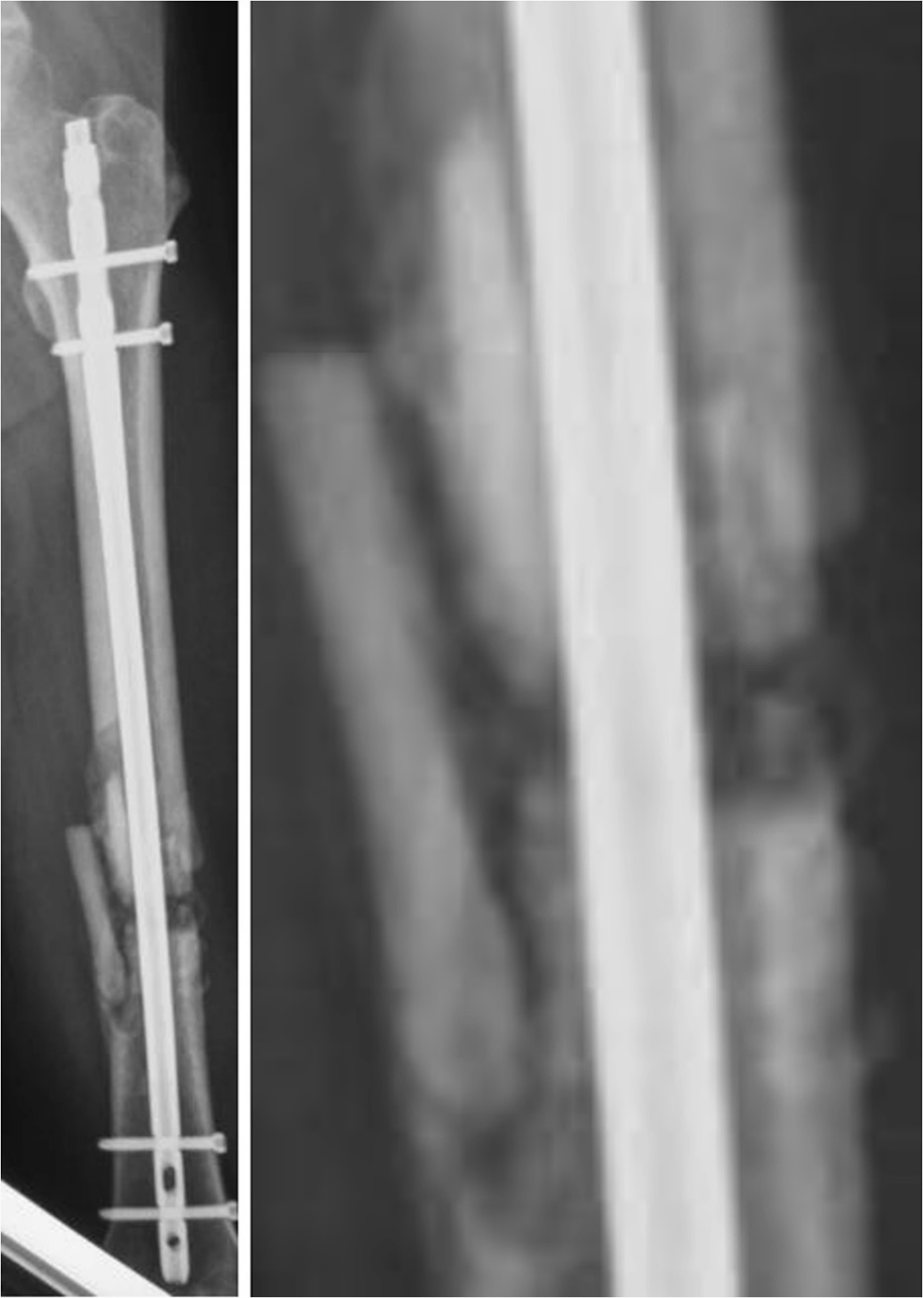
**Left, radiological image at 6 months, showing signs of nonunion, nondisplaced diaphysis, atrophic bone with gap.** Teriparatide was started at this point. Right, magnification of the image above.

**Figure 5:**
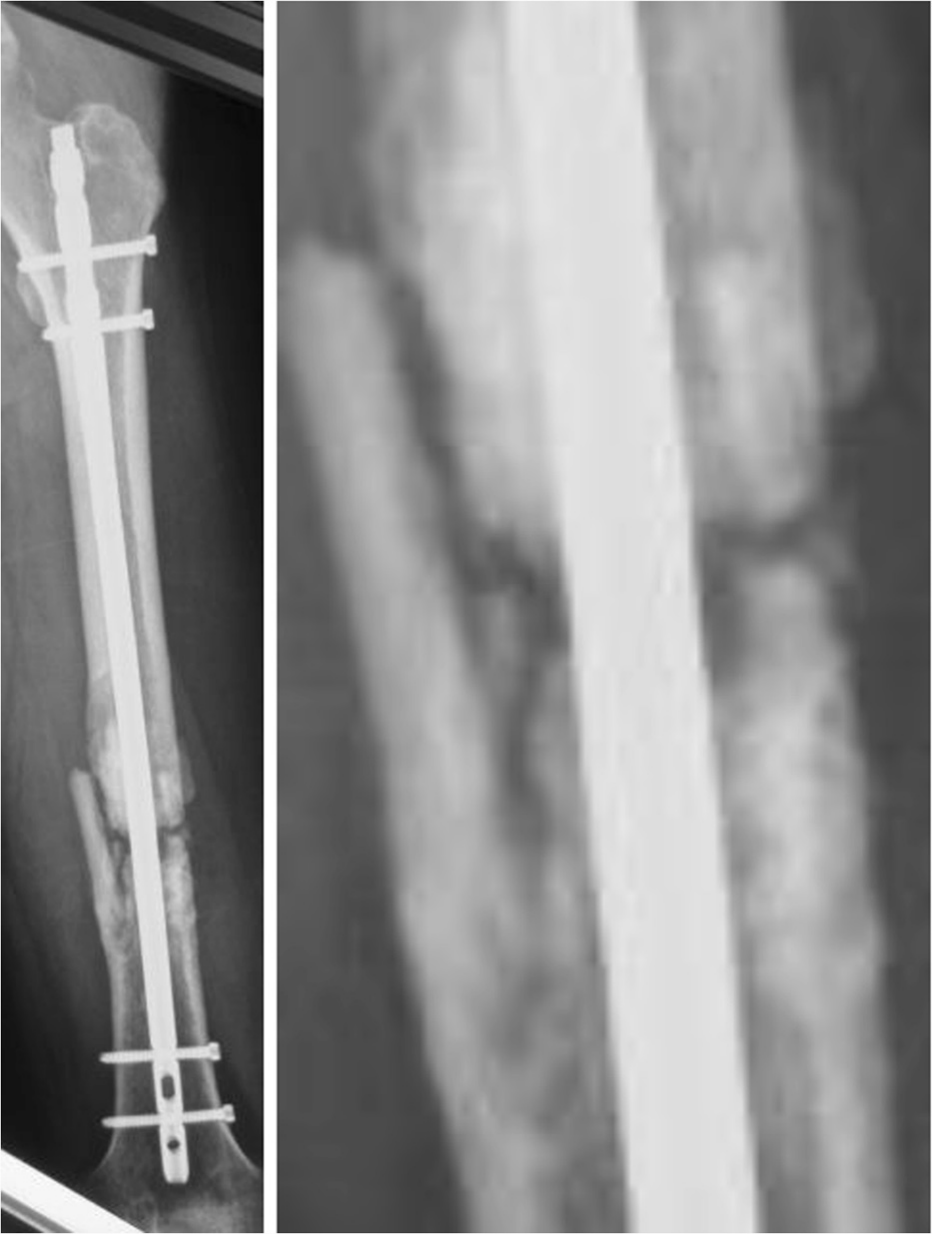
**Left, image obtained after 3 months of treatment with teriparatide.** Bone bridging and a decrease in interfragment gaps are observed. Right, magnification of the image above.

**Figure 6:**
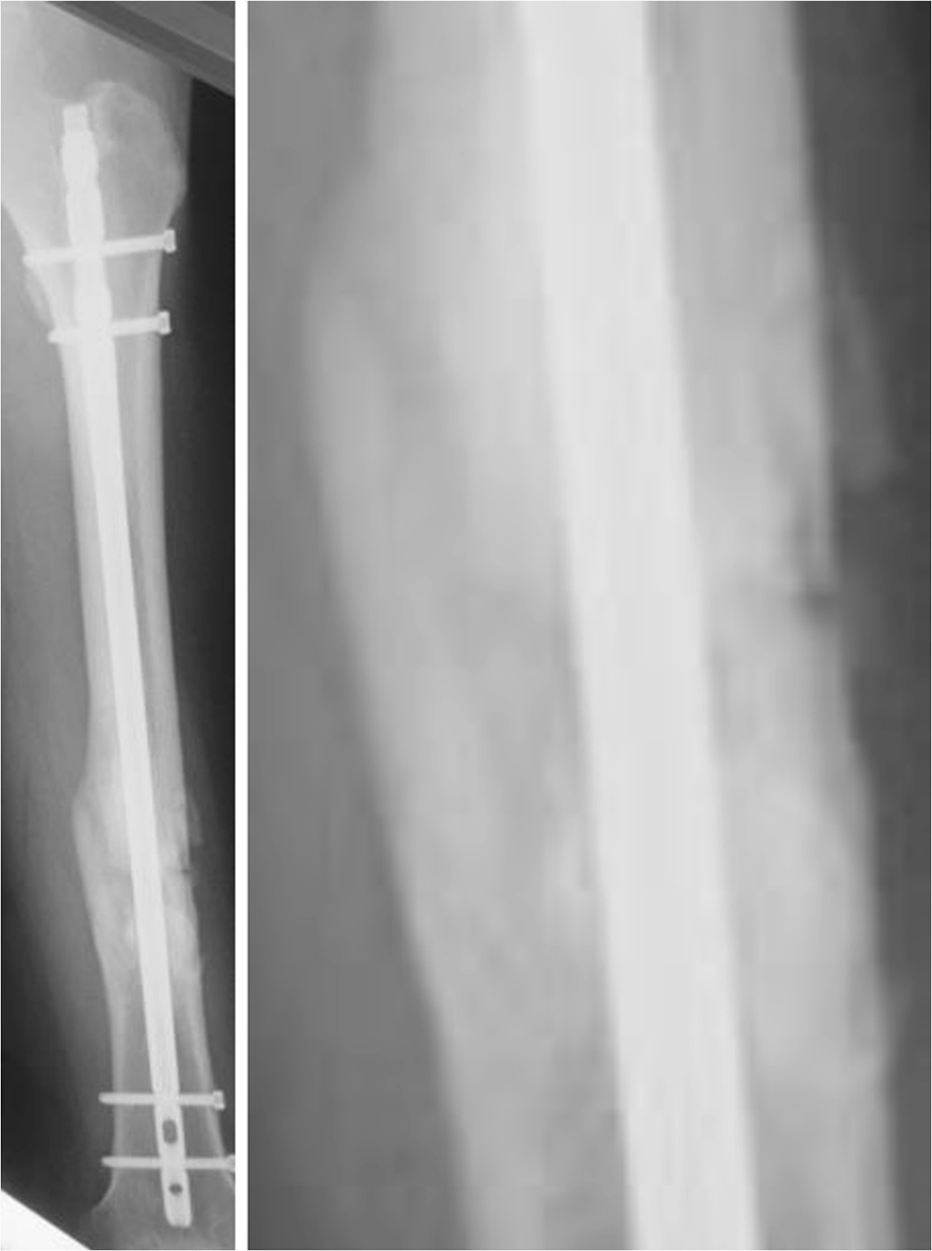
**Left, image obtained at 6 months showing healing of the nonunion.** Right, magnification of the image above.

A 56-year-old Japanese man was involved in a traffic accident. He was diagnosed with left diaphyseal femoral shaft fracture (OTA [Orthopaedic Trauma Association’s Fracture and Dislocation Compendium] 32-C1.3) (Figure 1) and was treated with intramedullary osteosynthesis nailing surgery at another hospital (Figure 2). Radiologic assessments at 3 months (Figure 3) did not show any sign of healing. This was consistent with the clinical manifestations of pain, movement at the fracture site, and left leg weakness. Physical examination and laboratory tests, including white blood cell counts, C-reactive protein, and erythrocyte sedimentation rate, were normal (Table 1), ruling out underlying infection. The patient denied smoking and alcohol abuse and had no history of metabolic disease or glucocorticoid intake. Other laboratory data, including serum alkaline phosphatase, intact PTH, and calcium, were normal (Table 1). However, he had high levels of bone quality marker, uncarboxylated osteocalcin ((ucOC), 18.8 ng/ml; reference value, <4.50 ng/ml) and serum homocysteine ((Hcys), 21.9 nmol/ml; reference value, 3.7 – 13.5 nmol/ml). Dual-energy X-ray absorptiometry assessment of bone density showed almost normal: 0.891 g/cm^2^ and T-score of -0.2 standard deviation on his radius. In other words, this patient had mildly compromised bone quality [5-8]. Callus formation in the fracture area remained poor 3 months after the first surgery, but the patient could not take any more time away from his company. Therefore, when he left the hospital he used crutches to assist with partial weight bearing ambulation. Both LIPUS and vitamin K2 preparation administrations were initiated at this time with the aim of lowering his high ucOC level. Callus formation in the fracture area was still insufficient at 3 months following initiation of LIPUS. Therefore, teriparatide was started in combination with LIPUS (Figure 4). Callus formation was good when assessed at 3 months after the start of teriparatide intervention (Figure 5). Bony union was good and full weight bearing was permitted after administration of teriparatide for 6 months (Figure 6). The ucOC levels improved to 5.6 ng/ml and serum Hcys improved to 10.8 nmol/ml 6 months after initiating teriparatide (Table 1).

**Table 1:** Laboratory findings

	**Normal value**	**Before teriparatide administration**	**After teriparatide administration**
BAP (μg/ml)	(3.7-20.9)	25.4	19.9
PINP (μg/ml)	(19.5-71.2)	37.1	48.0
DPD (nmol/mml･Cr)	(2.1-5.4)	18.7	6.9
Serum NTX (NMOLBCE/L)	(9.5-17.7)	17.6	16.6
TRACP-5B (μU/dl)	(170–590)	446	272
ucOC (ng/ml)	(<4.5)	18.8	5.6
Serum Hcys (nmol/ml)	(3.7-13.5)	21.9	10.9
Urine Pen (μg/mg･Cr)	(0.019-0.070)	0.042	0.023
intactPTH (pg/ml)	(10–65)	57	54
25-OHVitD (ng/ml)	(7–41)	7	10
Vit.B12 (pg/ml)	(180–914)	700	981
Vit.B6 (ng/ml)	(6–40)	7.2	15.2
Folate (ng/ml)	(>3.1)	2.0	4.2
Ca (mg/dL)	(8.4-9.7)	8.6	9.1
P (mg/dL)	(2.5-4.5)	2.9	2.7
Cr (mg/dL)	(0.6-1.2)	0.9	0.9
eGFR (ml/min/1.73 m^2^)	(>90)	98	97
CRP (mg/dL)	(<0.3)	0.0	0.0

*Abbreviation*; *BAP* Bone alkaline phosphatase, *PINP* procollagen typeIN-terminal propeptide, *DPD* dihydropyrimidine dehydrogenase, *NTX* N-terminal crosslinking telopeptide of typeIcollagen, *TRACP-5B* Tartrate-resistant Acid Phosphatase type 5B, *ucOC* uncarboxylated osteocalcin, *Hcys* homocysteine, *Pen* pentosidine, *PTH* parathyroid hormone, *25-OHVitD* 25-hydroxyvitamin-D, *Ca* calcium, *P* phosphorus, *Cr* creatinine, *CRP* C-reactive protein, *eGFR* estimated glomerular filtration rate.

## Conclusions

Intermittent administration of human parathyroid hormone (hPTH) has an anabolic effect on bone in humans and is expected to be a potent agent for fracture healing [3]. For nonunion, a second intervention will undoubtedly be necessary, carrying additional risks and potential complications as well as increases in healthcare costs. Therefore, any effective treatment that can solve this situation should be considered. Several recent studies have revealed that intermittent treatment with PTH stimulates osteogenesis in experimental fracture healing of cortical bones and that PTH effects on cortical bone repair are site-specific. Aspenberg *et al.*, in a prospective, randomized, double blind study of conservative fracture treatment for 102 postmenopausal women with distal radial fractures, showed than the time to healing was shorter in a teriparatide 20 mg group than in a placebo group [9]. Warden *et al. *[4] reported that teriparatide and LIPUS have contrasting additive, rather than synergistic, effects during fracture healing. Teriparatide primarily increased callus bone mineral content (BMC) without influencing its size, whereas LIPUS increased callus size without influencing BMC in rat studies. Fracture healing is a complex biologic process and is impacted by multiple factors [10]. Watanabe *et al. *[11] reported that LIPUS is a relatively new technique for the acceleration of fracture healing in nonunion situations. It has a frequency of 1.5 MHz, a signal burst width of 200 microseconds, a signal repetition frequency of 1 kHz, and an intensity of 30 mW/cm^2^. The beneficial effect of fracture healing acceleration with LIPUS is considered to be larger in patients with potentially negative factors for fracture healing. LIPUS is a pain-free therapy performed daily at home by the patient with the possibility of avoiding further surgical procedures. This outpatient treatment reduces the length of hospital stays and reduces overall system wide health care expenses. The incidence of delayed union and nonunion is 5% to 10% of all fractures. While LIPUS has beneficial effects on collagen enzymatic cross-link formation, mechanical stress may improve bone quality. Nonunion is a severe complication for the patient, which has a negative impact on quality of life. Treatment for nonunion typically requires a second surgical intervention to provide stability and to stimulate bone healing. These surgeries include locked intramedullary nailing, dynamic compression plating, external fixation with Ilizarov’s principles, and at times autografting. But Brinker *et al.* reported that all patients with nonunion who met their screening criteria should be referred to an endocrinologist for evaluation because they were likely to have undiagnosed metabolic or endocrine abnormalities that would interfere with bone healing [12]. Two interventions found to accelerate fracture healing processes are conbination of LIPUS and teriparatide without a second surgical intervention in this nonunion patient with deteriorated bone quality. No side effects occurred.

The case reported herein is a femoral shaft atrophic nonunion in which traditional interventions failed. This is the first reported case of diaphyseal nonunion with deterioration of bone quality in long bones resolved with teriparatide and LIPUS. The difference in teriparatide activity on trabecular and cortical bone suggests that teriparatide could accelerate healing in nonunions in diaphyseal nonunion in long bone with deterioration of bone quality. The authors are aware that this is just one case report; thus, new experimental studies and clinical trials with larger groups of subjects must be conducted in order to assess the efficacy of combination teriparatide and LIPUS intervention and the fracture healing.

## Consent

Written informed consent was obtained from the patient for publication of this Case report and any accompanying images. A copy of the written consent is available for review by the Editor-in-Chief of this journal.

## Competing interests

The authors declare that they have no competing interests.

## Authors’ contributions

KN performed the surgery. YS helped of the surgery, and helped to draft the manuscript. NM helped to draft the manuscript. SY helped of the surgery. MH helped to draft the manuscript. YK helped to draft the manuscript. HS helped to draft the manuscript. HK helped to draft the manuscript. All authors read and approved the final manuscript.

## References

[B1] Takikawa S Matsui N Kokubu T Tsunoda M Fujioka H Mizuno K Azuma Y Low intensity pulsed ultrasound initiates bone healing in rat nonunion fracture model J Ultrasound Med 2001 20 3 197 206 11270523 10.7863/jum.2001.20.3.197

[B2] Oteo-Alvaro A Moreno E Atrophic humeral shaft nonunion treated with teriparatide (rh PTH 1–34): a case report J Shoulder Elbow Surg 2010 19 7 22 28 10.1016/j.jse.2010.05.005 20846618

[B3] Nozaka K Miyakoshi N Kasukawa Y Maekawa S Noguchi H Shimada Y Intermittent administration of human parathyroid hormone enhances bone formation union at the site of cancellous bone osteotomy in normal and ovariectomized rats Bone 2008 42 1 90 97 10.1016/j.bone.2007.08.041 17997377

[B4] Warden SJ Komatsu DE Rydberg J Bond JL Hassett SM Recombinant human parathyroid hormone (PTH 1–34) and low-intensity pulsed ultrasound have contrasting additive effects during fracture healing Bone 2009 44 3 485 494 10.1016/j.bone.2008.11.007 19071238

[B5] Saito M Marumo K Ushiku C Kato S Sakai S Hayakawa N Mihara M Shiraishi A Effects of alfacalcidol on mechanical properties and collagen cross-links of the femoral diaphysis in glucocorticoid-treated rats Calcif Tissue Int 2011 88 4 314 324 10.1007/s00223-011-9472-6 21327766

[B6] Saito M Fujii K Marumo K Degree of mineralization-related collagen crosslinking in the femoral neck cancellous bone in cases of hip fracture and controls Calcif Tissue Int 2006 79 3 160 168 10.1007/s00223-006-0035-1 16969591

[B7] Saito M Marumo K Collagen cross-links as a determinant of bone quality: a possible explanation for bone fragility in aging, osteoporosis, and diabetes mellitus Osteoporos Int 2010 21 2 195 214 10.1007/s00198-009-1066-z 19760059

[B8] Saito M Marumo K Soshi S Kida Y Ushiku C Shinohara A Raloxifene ameliorates detrimental enzymatic and nonenzymatic collagen cross-links and bone strength in rabbits with hyperhomocysteinemia Osteoporos Int 2010 21 4 655 666 10.1007/s00198-009-0980-4 19484165

[B9] Aspenberg P Genant HK Johansson T Nino AJ See K Krohn K García-Hernández PA Recknor CP Einhorn TA Dalsky GP Mitlak BH Fierlinger A Lakshmanan MC Teriparatide for acceleration of fracture repair in humans: a prospective, randomized, double-blind study of 102 postmenopausal women with distal radial fractures J Bone Miner Res 2010 25 2 404 414 10.1359/jbmr.090731 19594305

[B10] Giannotti S Bottai V Dell'osso G Pini E De Paola G Bugelli G Guido G Current medical treatment strategies concerning fracture healing Clin Cases Miner Bone Metab 2013 10 2 116 120 24133528 PMC3796998

[B11] Watanabe Y Matsushita T Bhandari M Zdero R Schemitsch EH Ultrasound for fracture healing: current evidence J Orthop Trauma 2010 24 Suppl 1 56 61 10.1097/BOT.0b013e3181d2efaf 20182238

[B12] Brinker MR O'Connor DP Monla YT Earthman TP Metabolic and endocrine abnormalities in patients with nonunions J Orthop Trauma 2007 21 8 557 570 10.1097/BOT.0b013e31814d4dc6 17805023

